# Triglyceride-glucose index and mortality in congestive heart failure with diabetes: a machine learning predictive model

**DOI:** 10.3389/fendo.2025.1675152

**Published:** 2025-10-15

**Authors:** Lin Yu, Haizhu Chen, Jiwen Zhang, Wei Han

**Affiliations:** Department of Cardiovascular Medicine, Foshan Clinical Medical School of Guangzhou University of Chinese Medicine, Foshan, China

**Keywords:** triglyceride glucose index, congestive heart failure, diabetes, mortality, machine learning

## Abstract

**Background:**

The triglyceride-glucose (TyG) index serves as a marker for insulin resistance. Research exploring the link between the TyG index and adverse outcomes among patients suffering from congestive heart failure (CHF) and diabetes mellitus (DM) is limited. This investigation endeavors to uncover the connection between the TyG index and mortality risk in subjects suffering from CHF and DM.

**Methods:**

We obtained clinical data for patients with CHF and DM from the MIMIC-IV (3.1) database. The optimal cutoff value for the TyG index was determined using X-tile software, and patients were classified into three groups. The primary outcome was 28-day hospital mortality, and the secondary outcome was 28-day ICU mortality. We used restricted cubic splines (RCS), COX regression analysis, and Kaplan-Meier survival curves to examine the association between the TyG index and adverse outcomes. Subgroup analyses were conducted based on age, gender, chronic pulmonary disease, atrial fibrillation, hypertension, and mechanical ventilation to assess the robustness of our findings. Feature selection was performed using LASSO regression, and predictive modeling was carried out using machine learning algorithms.

**Results:**

This study included 1046 patients with CHF and DM. Using a fully adjusted COX regression model, we identified a significant association between the TyG index and both 28-day hospital mortality (HR=1.31, 95% CI: 1.09–1.57, P=0.004) and 28-day ICU mortality (HR=1.29, 95% CI: 1.07–1.54, P=0.006). Using restricted cubic spline analysis, a linear link between the TyG index and mortality rates was found, indicating that a rise in TyG correlates with a heightened risk of unfavorable outcomes. The predictive performance was evaluated using seven machine learning algorithms, with the Random Survival Forest (RSF) algorithm achieving the best performance (AUC=0.817).

**Conclusions:**

In patients with CHF and DM, TyG exhibited a linear correlation with both 28-day hospital mortality and 28-day ICU mortality. Elevated TyG values were significantly linked to a heightened risk of adverse events.

## Introduction

Congestive heart failure (CHF) is a pathological state. In this state, the heart can’t sustain adequate cardiac output to fulfill the body’s metabolic demands. It ranks as a significant factor in the global rise of illness and death rates (1–4). With demographic changes, advances in medical therapy, and increased incidence of concomitant diseases such as diabetes, the epidemiologic profile of CHF has been affected accordingly (2, 3). Research in epidemiology has established a notable link connecting diabetes mellitus(DM) and heart failure(HF) (5, 6). Type 2 diabetes mellitus (T2DM) significantly contributes to the risk of cardiovascular events. CHF is a prime example. This condition heightens the risk of sickness and fatality for those affected (7). Numerous studies have consistently demonstrated that HF patients with DM experience a more adverse prognosis. For instance, a health insurance study revealed that the mortality rate for patients with both T2DM and HF is as high as 32.7 per 1000 person-years. In contrast, it is significantly lower at 3.7 per 1000 person-years for those with HF but normal blood glucose levels (8). Furthermore, among patients with HF and reduced ejection fraction (HFrEF), those with diabetes experience a significantly higher mortality risk than their non-diabetic counterparts (9).

The triglyceride-glucose (TyG) index merges indicators of triglycerides(TG) and fasting blood glucose(FBG). It helps assess insulin sensitivity in the body. It acts as an alternative measure of insulin resistance(IR) (10). The pathophysiological state termed IR is defined by a decreased capacity of the body to respond to and metabolize insulin, culminating in hyperglycemia (11). In HF patients with DM, IR and metabolic disturbances are exacerbated, leading to myocardial insulin resistance and mitochondrial dysfunction, which profoundly impact myocardial energy metabolism and cardiac function (12). Hence, a deeper exploration into the role of IR in these patients is highly warranted. Adverse cardiovascular and metabolic events have been found to be linked to the TyG index (13–16). Despite ongoing research, the specific link connecting the TyG index and death among people with CHF and DM remains elusive. IR, a pivotal driver in the progression of CHF and DM, has been extensively studied. However, further exploration is necessary to delineate the forecasting power of the TyG index concerning fatality rates among this patient population.

The relation connecting the TyG index and fatality in CHF and DM was the focus of this study. It is anticipated that this investigation will furnish vital information concerning the relevance of the TyG index in prognosis for these people and may provide a novel approach to risk stratification and management.

## Methods

### Data source

This research leveraged information from MIMIC-IV 3.1, an open-access intensive care repository encompassing detailed medical notes for over 300,000 patients treated at Beth Israel Deaconess Medical Center (BIDMC) from 2008 to 2022. The BIDMC’s Institutional Review Committee sanctioned the utilization of these research materials and dispensed with the need for informed consent. The researchers then accessed the database using certificate number 13874481.

### Study population

**Figure 1:**
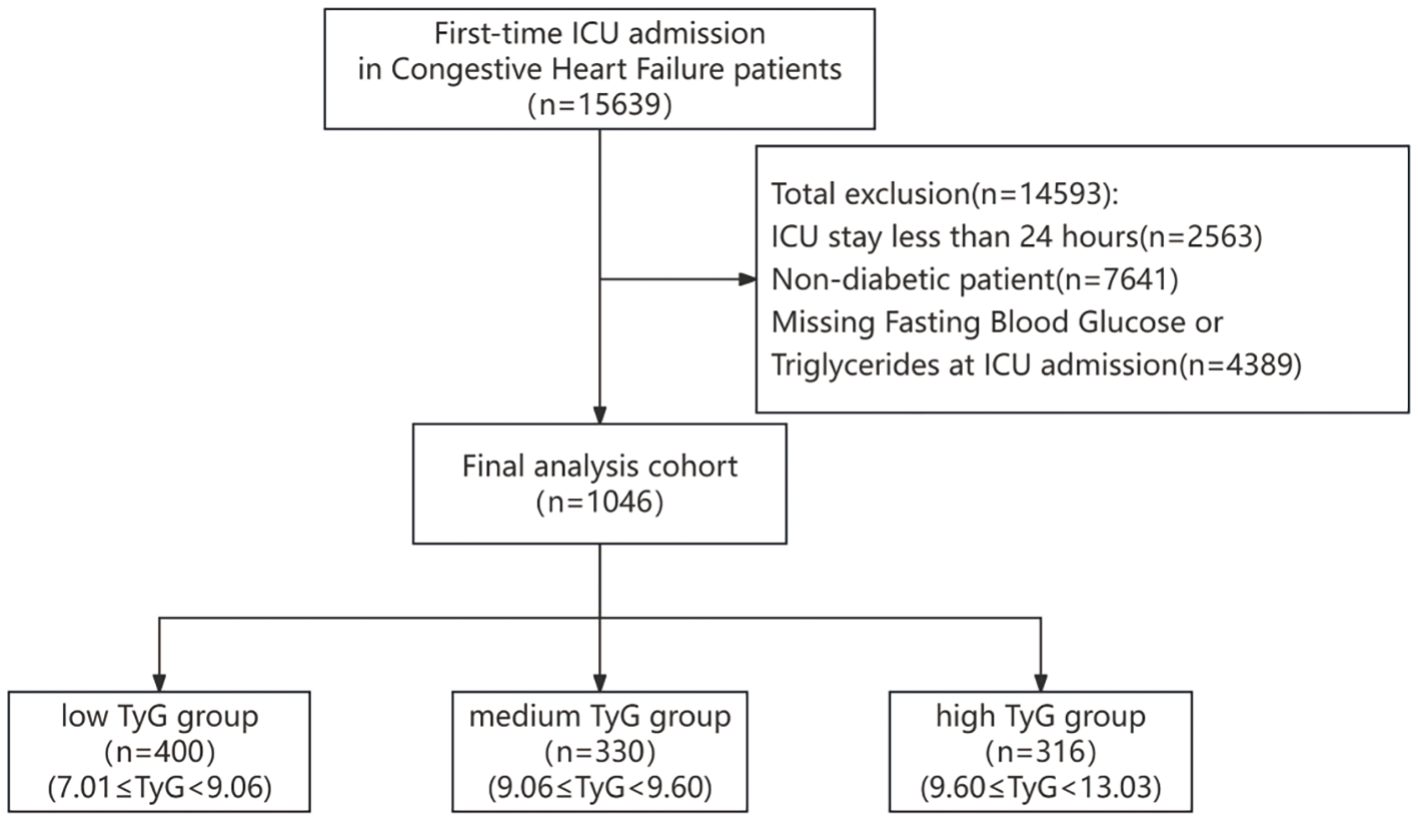
Patient selection flowchart.

Patients admitted to the ICU with congestive heart failure were recognized via ICD-9 and ICD-10 codes and included within the research. If a patient had repeated ICU admissions, only the initial admission was chosen for analysis. The exclusion criteria for patients, as illustrated in **Figure 1**, are detailed henceforth: (1) ICU stays lasting fewer than 24 hours (n=2563); (2) patients without diabetes (n=7641); (3) absence of data on fasting blood glucose and triglyceride levels (n=4389).

### Outcome

The main outcome was 28-day hospital mortality, evaluated from the day of admission and monitored for 28 days to assess survival status. This indicator comprehensively reflects the mortality risk of patients throughout their hospital stay, including treatment outcomes in both the ICU and other wards. The secondary outcome was 28-day ICU mortality, ascertained from the day of ICU admission and tracked for survival over the following 28 days. This indicator more specifically reflects the treatment outcomes within the ICU and the severity of the patients’ conditions.

### Data collection

Data collection was done utilizing PostgresSQL (version 17.1) and Navicate Premium (version 17.0.8) software. During the initial 24 hours of ICU admission, we collected extensive data. The patient data retrieved included information on age, gender, ethnicity, and weight. We also gathered vital signs, which included heart rate, respiratory rate(RR), systolic and diastolic blood pressure(SBP, DBP), temperature, and arterial oxygen saturation(Spo2). The disease severity metrics that were collected consisted of sepsis-organ failure assessment score (SOFA), Oxford Acute Severity of Illness Score (OASIS), Glasgow Coma Scale (GCS), and Charlson Comorbidity Index (CCI). Information on comorbid conditions such as hypertension, coronary artery disease(CAD), atrial fibrillation(AF), chronic pulmonary disease, acute kidney injury(AKI) and sepsis was gathered using ICD codes. The laboratory values that were retrieved included red blood cell count (RBC), white blood cell count (WBC), platelet count (PLT), triglycerides (TG), fasting blood glucose (FBG), serum sodium, serum potassium, serum calcium, blood urea nitrogen(BUN), serum creatinine(Cr), prothrombin time International Normalized Ratio (INR), Prothrombin Time (PT), Partial Thromboplastin Time (PTT), and urine output. The medications that were included for extraction were angiotensin-converting enzyme inhibitors (ACEI)/angiotensin receptor blockers (ARB), beta-blockers, insulin, and statins. The therapeutic interventions that were extracted included renal replacement therapy (CRRT) and mechanical ventilation (MV). The triglyceride glucose index (TyG) is calculated using the following formula: TyG=ln [triglyceride (TG in mg/dl) × fasting blood glucose (FBG in mg/dl)/2] (17). In this study, we obtained the levels of TG and FBG from patients on the first day of ICU admission to calculate the ICU admission TyG index.

### Statistical analysis

The analysis excluded variables that had a rate of missing data exceeding 20%, while those with a rate under 20% underwent multiple imputations. Using X-tile software, the optimal cutoff value for TyG was identified (18). X-tile software was employed to determine the optimal cutoff value using an enumeration method. This method systematically evaluates all potential cutoff values through statistical testing and selects the one with the smallest P value as optimal (19). Based on the identified cutoff values, the study population was stratified into low, medium, and high TyG groups. Continuous variables conforming to a normal distribution were represented by means (standard deviations [SDs]). Variance analysis (ANOVA) was applied to evaluate these variables. We analyzed variables that did not adhere to a normal distribution using the Mann–Whitney U test or Kruskal–Wallis test. We displayed categorical variables as counts and percentages. We assessed them using the χ² test or Fisher’s exact test. The log-rank test was used alongside Kaplan-Meier survival curves to compare 28-day survival rates among the three groups. Cox proportional hazard regression models were employed to calculate the hazard ratio(HR) and 95% Confidence Interval(CI) for event occurrence. Drawing on the outcomes of univariate Cox regression analysis and our clinical expertise, we refined the covariates in Model I and Model II. Model I accounted for gender, race, age, and weight. Model II further incorporated adjustments for SBP, HR, temperature, AF, AKI, ACEI/ARB, beta-blockers, insulin, statin, MV, SOFA, WBC, BUN, and INR. A two-tailed P value less than 0.05 served to ascertain statistical significance. R software (version 4.4.2) was employed to conduct all statistical analyses.

### Restricted cubic spline

For this investigation, we assembled a comprehensive dataset including survival data as the outcome variable. The TyG index was employed as an uninterrupted predictor. We also collected a range of covariates, such as gender, race, age, weight, SBP, HR, temperature, AF, AKI, ACEI/ARB, beta-blockers, insulin, statin, MV, SOFA, WBC, BUN, and INR. We wanted to explore the possible nonlinear connection linking changes in the TyG index and death. So, we utilized a Cox regression model with restricted cubic splines (RCS).

### Subgroup analysis

We executed subgroup and multivariate analyses in accordance with predetermined criteria, such as age, gender, chronic pulmonary disease, AF, hypertension, and MV. During the multivariate analyses, we accounted for the following covariates: gender, race, age, weight, SBP, HR, temperature, AF, AKI, ACEI/ARB, beta-blockers, insulin, statin, MV, SOFA, WBC, BUN, and INR. For the multivariate analysis, conducted separately for male and female subgroups, gender was not included as an adjustment factor. Patients were categorized into two age categories: under 65 years and 65 years or older. We conducted Cox proportional hazard regression analysis within each category. The findings were visualized via forest plots that highlighted the HR and its 95% CI.

### Establishment and validation of the prediction models

The dataset undergoes five-fold cross-validation sampling, splitting it into a training set and an internal validation set. When there are numerous features, feature selection is conducted using the Lasso method. Lasso incorporates L1 regularization to select features, reduce dimensionality, and eliminate redundant features by compressing coefficients.

Suitable variables were integrated into the machine learning framework. The dataset was partitioned into training and validation subsets in a 7:3 distribution. To predict the 28-day in-hospital mortality risk in patients with CHF complicated by DM, we individually analyzed the screened variables using the following methods: Cox Proportional Hazards Model (CoxPH), Classification and Regression Trees (CART), Gradient Boosting Machine (GBM), Elastic Network (ENet), Neural Network (NN), Random Survival Forest (RSF), and Extreme Gradient Boosting (XGBoost). Hyper-parameter tuning was conducted during the model-building process. The training subset was employed to construct the model, while the validation subset was employed to assess its effectiveness. Model performance was assessed using the receiver operating characteristic curve (ROC) and its area under the curve (AUC). We used decision curve analysis (DCA) to assess clinical validity. And we employed calibration curves to evaluate the model’s accuracy in predicting absolute risk.

## Results

### Baseline characteristics

The baseline features of 1046 individuals diagnosed with CHF and DM were derived from the MIMIC-IV database (**Table 1**). It should be emphasized that every patient involved in this study was admitted to the ICU. The distribution of missing data for each variable is illustrated in **Supplementary Figure S1**. The essential features of the participants are described in **Table 1**. The study cohort was predominantly male (60%) and featured a high rate of comorbid conditions, including CAD(70%), hypertension (86%), AKI(89%), and sepsis (60%). By employing X-tile software, the optimal threshold for the TyG index was established, leading to the categorization of participants into three distinct groups: low TyG (7.01≤TyG<9.06, n=400), medium TyG (9.06≤TyG<9.60, n=330), and high TyG (9.60≤TyG<13.03, n=316). Individuals in the high TyG group tended to be younger, with higher weight, heart rate, RR, and temperature, but lower Spo2. Compared with other groups, they had lower OASIS and CCI scores, a decreased occurrence of AF, and a higher prevalence of sepsis. In terms of laboratory findings, they exhibited elevated WBC, TG, FBG, BUN, and serum creatinine levels, but lower blood calcium and PT, along with shorter PTT. Regarding therapeutic procedures, a higher proportion underwent CRRT, while fewer required Mechanical ventilation.

**Table 1:** Baseline characteristics.

Characteristic	Overall N=1,046	Low TyG group N=400	medium TyG group N=330	High TyG group N=316	P value
Age (year)	70 (62,79)	73 (64, 80)	72 (63,80)	66 (58,75)	**<0.001**
Gender (%)					0.875
female	420 (40%)	160 (40%)	136 (41%)	124 (39%)	
male	626 (60%)	240 (60%)	194 (59%)	192 (61%)	
Race (%)					0.070
white	670 (64%)	247 (62%)	228 (69%)	195 (62%)	
Other	376 (36%)	153 (38%)	102 (31%)	121 (38%)	
Weight (Kg)	87 (73, 103)	83 (67,99)	88 (73, 103)	92 (78,112)	**<0.001**
Heart rate(bmp)	87 (75,101)	86 (74,98)	85 (74,101)	90 (78, 104)	**0.003**
RR(bmp)	20(16,24)	18(16,22)	20(16,24)	21(17,25)	**<0.001**
SBP(mmHg)	123 (107, 143)	120 (106, 141)	125 (108, 145)	126 (108, 146)	0.152
DBP(mmHg)	66 (55,79)	65 (53,79)	65 (54,79)	68 (57,80)	0.055
Temperature(°C)	36.7 (36.4, 37.1)	36.6 (36.3, 36.9)	36.7 (36.4, 37.0)	36.9 (36.5, 37.33)	**<0.001**
Spo2(%)	98 (95,100)	98 (95,100)	98 (95,100)	97 (94,99)	**<0.001**
SOFA	1(0,3)	1(0,3)	1(0,4)	2(0,4)	0.733
GCS	15(15,15)	15(15,15)	15(15,15)	15(15,15)	0.068
OASIS	34(28,39)	33 (26,38)	34(28,38)	35(30,42)	**<0.001**
CCI	8(6,10)	8(6,10)	8(6,10)	7(5,9)	**<0.001**
Chronic pulmonary disease, n (%)	344 (33%)	129 (32%)	112 (34%)	103 (33%)	0.882
Coronary heart disease, n (%)	736 (70%)	285 (71%)	243 (74%)	208 (66%)	0.083
Atrial fibrillation, n (%)	446 (43%)	183 (46%)	158 (48%)	105 (33%)	**<0.001**
Hypertension, n (%)	904 (86%)	338 (85%)	293 (89%)	273 (86%)	0.242
AKI, n (%)	932 (89%)	345 (86%)	298 (90%)	289 (91%)	0.060
Sepsis, n (%)	626 (60%)	190 (48%)	208 (63%)	228 (72%)	**<0.001**
RBC(10^9^/L)	3.41 (2.87,4.05)	3.35 (2.85,4.01)	3.37 (2.88, 3.93)	3.51 (2.93, 4.16)	0.153
WBC(10^9^/L)	10.0 (7.5, 13.1)	9.4 (6.9, 12.2)	10.2 (7.6, 13.1)	10.6 (8.0, 14.5)	**<0.001**
Platelet (10^9^/L)	186 (137, 246)	184 (130, 240)	186 (141, 249)	194 (140, 249)	0.396
TG(mg/dL)	126 (86, 191)	80 (66,99)	129 (110, 156)	257 (194,367)	**<0.001**
FBG(mg/dL)	166 (136, 201)	141 (120, 166)	170 (147, 199)	201(168,234)	**<0.001**
Sodium (mmol/L)	137 (134, 140)	137 (134, 140)	137 (134, 140)	137 (134, 139)	0.860
Potassium (mmol/L)	4.00 (3.60,4.40)	4.00 (3.70,4.40)	4.00 (3.70,4.40)	3.90 (3.60, 4.30)	0.224
Calcium (mg/dL)	8.30 (7.90, 8.80)	8.40 (7.90, 8.95)	8.30 (7.80, 8.80)	8.20 (7.70, 8.75)	**0.003**
BUN(mg/dL)	27(17,44)	24(16,40)	28(18,45)	28(17,45)	**0.007**
creatinine (mg/dL)	1.30 (0.90,2.10)	1.20 (0.80,1.80)	1.40 (0.90,2.20)	1.35 (1.00,2.30)	**0.002**
INR	1.20 (1.10, 1.40)	1.20 (1.10, 1.40)	1.20 (1.10,1.50)	1.20 (1.10, 1.40)	**0.016**
PT(S)	13.6 (12.4, 15.8)	13.6 (12.6, 15.9)	13.9 (12.6, 16.3)	13.3 (12.0, 15.0)	**<0.001**
PTT(S)	30(27,37)	31(27,38)	30(27,37)	30(27,34)	**0.004**
Urine output(mL)	1,460 (780,2,425)	1,350 (750,2,351)	1,466 (805,2,495)	1,554 (833,2,570)	0.340
ACEI/ARB, n (%)	384 (37%)	156 (39%)	109 (33%)	119 (38%)	0.229
Beta blockers, n (%)	772 (74%)	305 (76%)	237 (72%)	230 (73%)	0.353
Insulin, n (%)	940 (90%)	338 (85%)	302 (92%)	300 (95%)	**<0.001**
Statins, n (%)	666 (64%)	250 (63%)	208 (63%)	208 (66%)	0.629
CRRT, n (%)	151 (14%)	36 (9.0%)	49 (15%)	66 (21%)	**<0.001**
Mechanical ventilation, n (%)	928 (89%)	342 (86%)	293 (89%)	293 (93%)	**0.010**
Los hospital (days)	12(7,20)	12(7,17)	11(7,20)	14(8,23)	**0.006**
Los ICU (days)	4(2,9)	4(2,7)	4(2,8)	6(3,12)	**<0.001**
Hospital Mortality, n (%)	205 (20%)	58 (15%)	67 (20%)	80 (25%)	**0.001**
ICU Mortality, n (%)	150 (14%)	42 (11%)	48 (15%)	60 (19%)	**0.006**
28-day hospital Mortality, n (%)	216 (21%)	67 (17%)	70 (21%)	79 (25%)	**0.024**
28-day ICU Mortality, n (%)	228 (22%)	72 (18%)	74 (22%)	82 (26%)	**0.036**

RR, Respiratory Rate; SBP, Systolic blood pressure; DBP, Diastolic blood pressure; SpO2, Oxygen saturation; SOFA, Sequential organ failure assessment; GCS, Glasgow Coma Scale; OASIS, Oxford Acute Severity of Illness Score; CCI, Charlson Comorbidity Index; AKI, Acute kidney injury; WBC, White blood cell count; RBC, Red blood cell count; Platelet, Platelet count; TG, triglycerides; FBG, fasting blood glucose; BUN, Blood urea nitrogen; INR, International normalized ratio; PT, Prothrombin Time; PTT, Partial Thromboplastin Time; ACEI/ARB, Angiotensin-converting enzyme inhibitors/Angiotensin receptor blockers; CRRT, Continuous renal replacement therapy. Bolded values indicate statistical significance.

### Survival analysis

**Figure 2:**
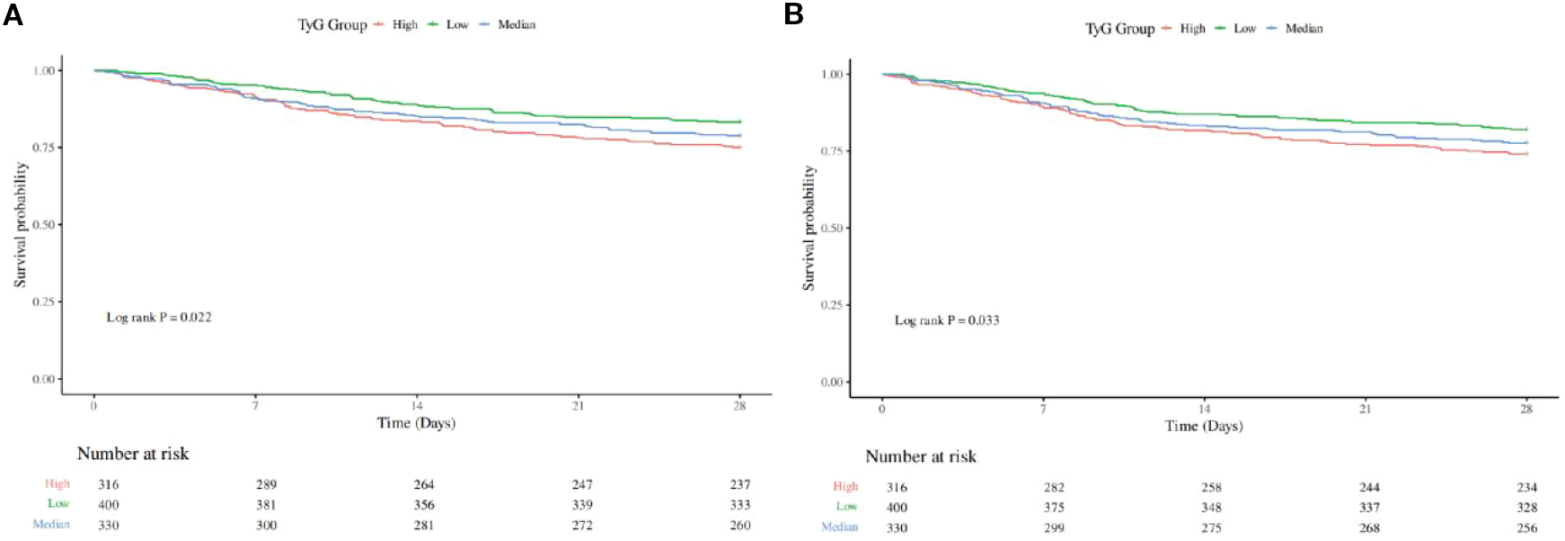
Kaplan-Meier survival analysis curve for all-cause mortality: **(A)** showing comparison of 28-Day Hospitalized Mortality between groups, **(B)** showing comparison of 28-Day ICU Mortality between groups.

To assess the frequency of the primary outcome across diverse groups, Kaplan-Meier survival curves were utilized (**Figure 2**). The high TyG group demonstrated a markedly higher 28-day hospital mortality rate than other groups (P=0.022) (**Figure 2A**). Likewise, The 28-day ICU mortality rate was significantly elevated in the high TyG group compared with other groups (P=0.033) (**Figure 2B**). These results imply that a high TyG index is tied to negative survival outcomes in individuals with diabetes and congestive heart failure.

### Association between mortality and TyG

This investigation revealed a robust link linking the TyG index and the likelihood for 28-day hospital mortality, which remained consistent across various models. when considered as a continuous measure, the TyG index demonstrated significant associations in the unadjusted model [HR=1.26, 95% CI: 1.06-1.50, P=0.008], Model I [HR=1.49, 95% CI: 1.25-1.78, P < 0.001], and Model II [HR=1.31, 95% CI: 1.09-1.57, P=0.004]. Similarly, the connection linking the TyG index and the likelihood of 28-day ICU mortality was consistent across different models. The adjusted model [HR=1.24, 95% CI: 1.05-1.47, P=0.012], Model I [HR=1.48, 95% CI: 1.24-1.76, P < 0.001], and Model II [HR=1.29, 95% CI: 1.07-1.54, P=0.006] (**Table 2**) also showed significant results (**Table 2**).

**Table 2:** Cox regression model.

TyG	Non-adjusted		Adjust I		Adjust II	
	HR (95% CI)	P value	HR (95% CI)	P value	HR (95% CI)	P value
28-day hospital mortality						
TyG continuous variable	1.26(1.06,1.50)	**0.008**	1.49(1.25,1.78)	**<0.001**	1.31(1.09,1.57)	**0.004**
TyG categorical variables						
Low TyG (7.01≤TyG<9.06)	ref		ref		ref	
Medium TyG (9.06 ≤ TyG < 9.60)	1.31(0.94,1.83)	0.113	1.41(1.01,1.98)	**0.044**	1.17(0.83,1.65)	0.365
High TyG (9.60≤TyG<13.03)	1.58(1.14,2.18)	**0.006**	2.10(1.50,2.93)	**<0.001**	1.77(1.25,2.51)	**0.001**
TyG group trend		**0.006**		**<0.001**		**0.002**
28-day ICU mortality						
TyG continuous variable	1.24(1.05,1.47)	**0.012**	1.48(1.24,1.76)	**<0.001**	1.29(1.07,1.54)	**0.006**
TyG categorical variables						
Low TyG (7.01≤TyG<9.06)	ref		ref		ref	
Medium TyG (9.06 ≤ TyG < 9.60)	1.29(0.93,1.78)	0.128	1.38(1.00,1.92)	0.051	1.12(0.80,1.56)	0.509
High TyG (9.60≤TyG<13.03)	1.52(1.11,2.09)	**0.010**	2.03(1.46,2.81)	**<0.001**	1.69(1.21,2.38)	**0.002**
TyG group trend		**0.009**		**<0.001**		**0.003**

HR, Hazard Ratio; CI, Confidence Interval.

Adjust I: Adjust: Gender, Race, Age, Weight.

Adjust II: Adjust: Gender, Race, AF, AKI, ACEI/ARB, beta-blockers, insulin, Statins, MV, Age, Weight, SBP, RR, Temperature, SOFA, WBC, BUN, INR.

AF, Atrial Fibrillation; AKI, Acute kidney injury; ACEI/ARB, Angiotensin-converting enzyme inhibitors/Angiotensin receptor blockers; MV, Mechanical Ventilation; SBP, Systolic blood pressure; DBP, Diastolic blood pressure; RR, Respiratory Rate; SOFA, Sequential organ failure assessment; WBC, White blood cell count; BUN, Blood urea nitrogen; INR, International normalized ratio. Bolded values indicate statistical significance.

When the TyG index was analyzed categorically, a strong connection linking the high TyG category and 28-day hospital mortality was observed. They also had a strong relationship with the 28-day ICU fatality rate across the three models.

### Restricted cubic spline

**Figure 3:**
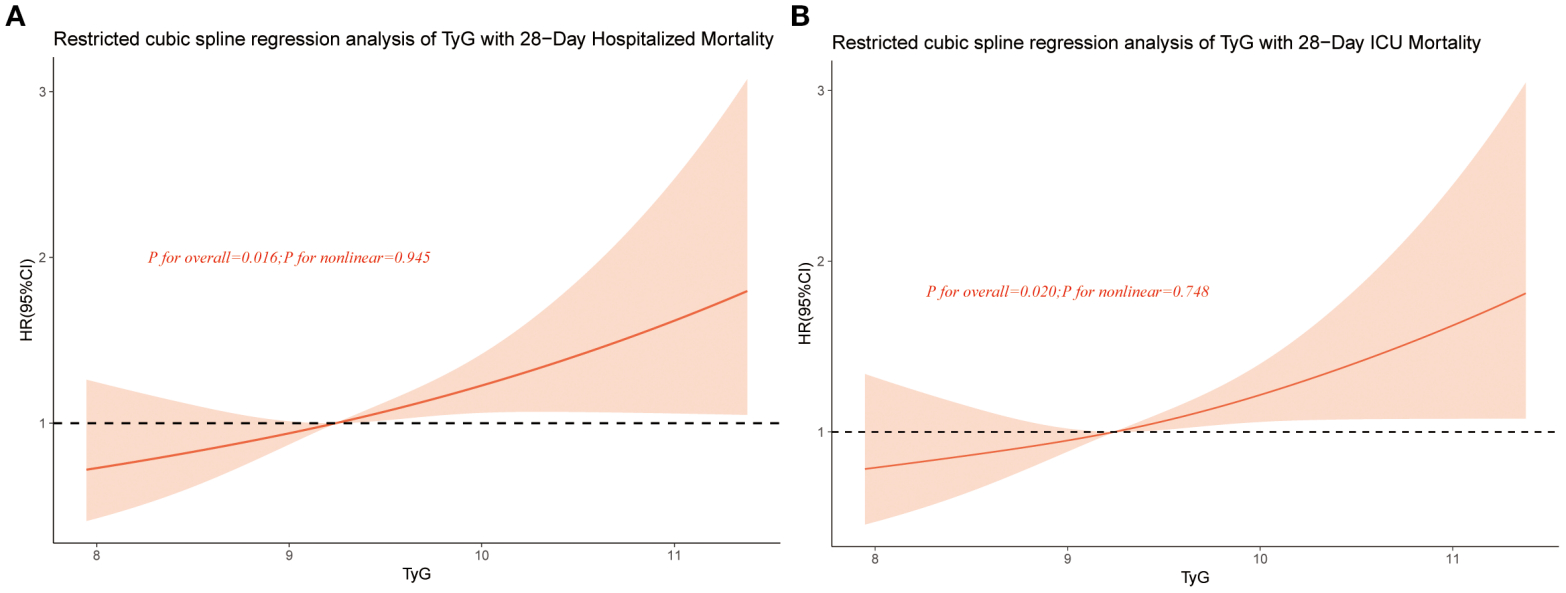
Restricted cubic spline curve for TyG hazard ratio. Red lines indicate fully adjusted risk ratios, shaded areas indicate 95% confidence intervals, and horizontal dashed hazard ratio. **(A)** Restricted cubic spline curve for 28-Day Hospitalized Mortality, **(B)** Restricted cubic spline curve for 28-Day ICU Mortality.

The RCS analysis was adjusted for potential confounding factors including gender, age, weight, SBP, HR, temperature, AF,AKI, ACEI/ARB, beta-blockers, insulin, statin, MV, SOFA, WBC, BUN, and INR. The RCS analysis for 28-day all-cause in-hospital mortality (**Figure 3A**) and 28-day all-cause ICU mortality (**Figure 3B**) both demonstrated a linear association between TyG and mortality risk.

### Subgroup analysis

**Figure 4:**
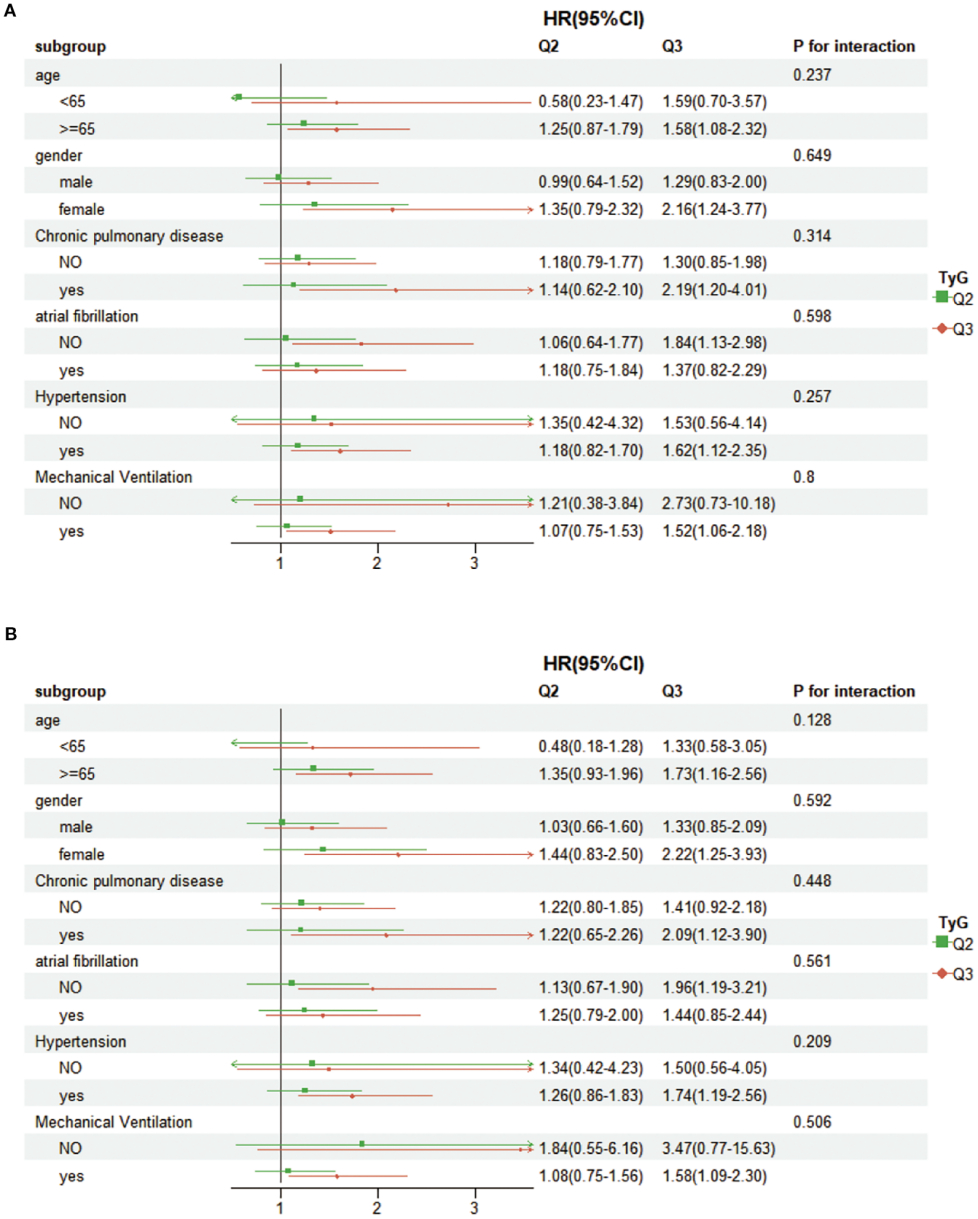
Forest plots showing subgroup analyses for **(A)** 28-day hospital mortality, **(B)** 28-day ICU mortality.

We performed subgroup analyses categorized by age (less than 65 years, 65 years and older), gender, chronic lung disease, atrial fibrillation, hypertension, and mechanical ventilation. The findings (**Figure 4**) showed no significant correlation linking TyG and any of the subgroups (interaction p-value > 0.05 across all subgroups). These deductions dedicate that the association of TyG with 28-day hospital fatality is uniform. It is also uniform for 28-day ICU mortality across different demographic groups.

### Feature selection

**Figure 5:**
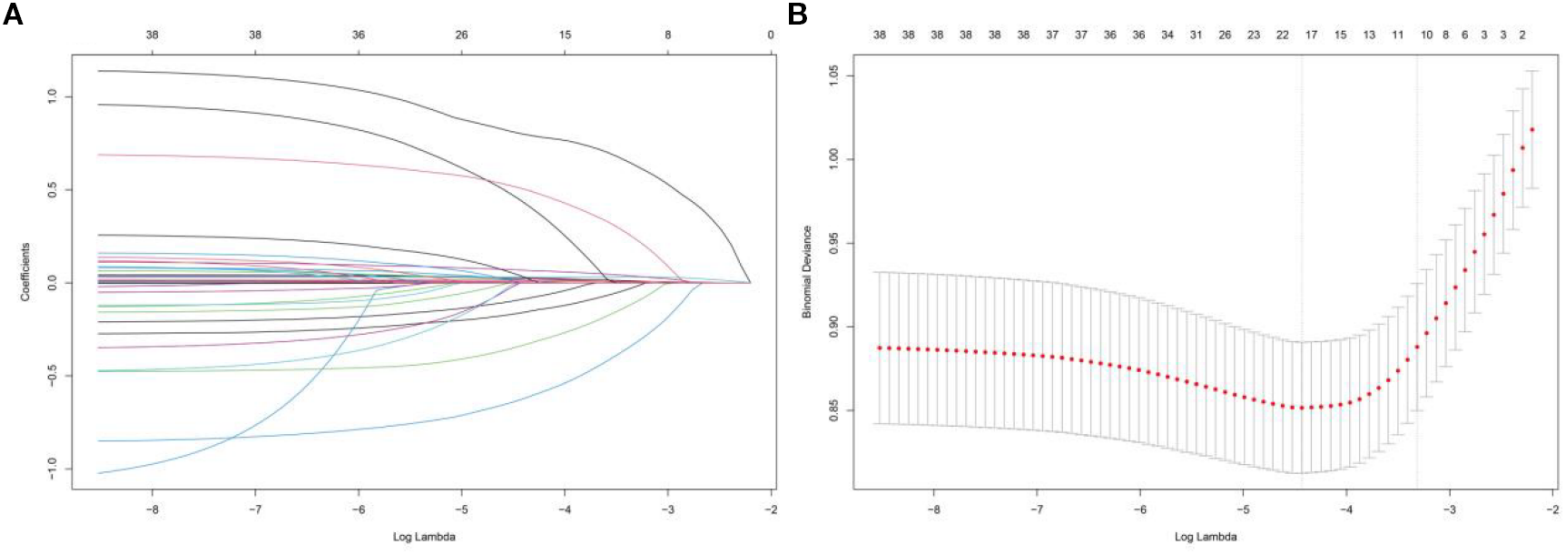
Lasso regression-based variable screening, **(A)** Characteristics of variable coefficient variations, **(B)** The process of selecting the optimal value of the parameter λ in the lasso regression model is carried out by the cross-validation method.

Lasso regression was applied to the training set to identify correlated features, with the variable coefficients shown in **Figure 5A**. A tenfold cross-validation method was employed to conduct iterative analyses (**Figure 5B**). Age, RR, temperature, SOFA, GCS, OASIS, CCI, AKI, sepsis, WBC, RBC, TyG, blood calcium, BUN, PTT, ACEI/ARB, beta-blockers, and CRRT were among the 18 variables that showed a strong link with 28-day hospital mortality.

### Predictive modeling and validation

**Figure 6:**
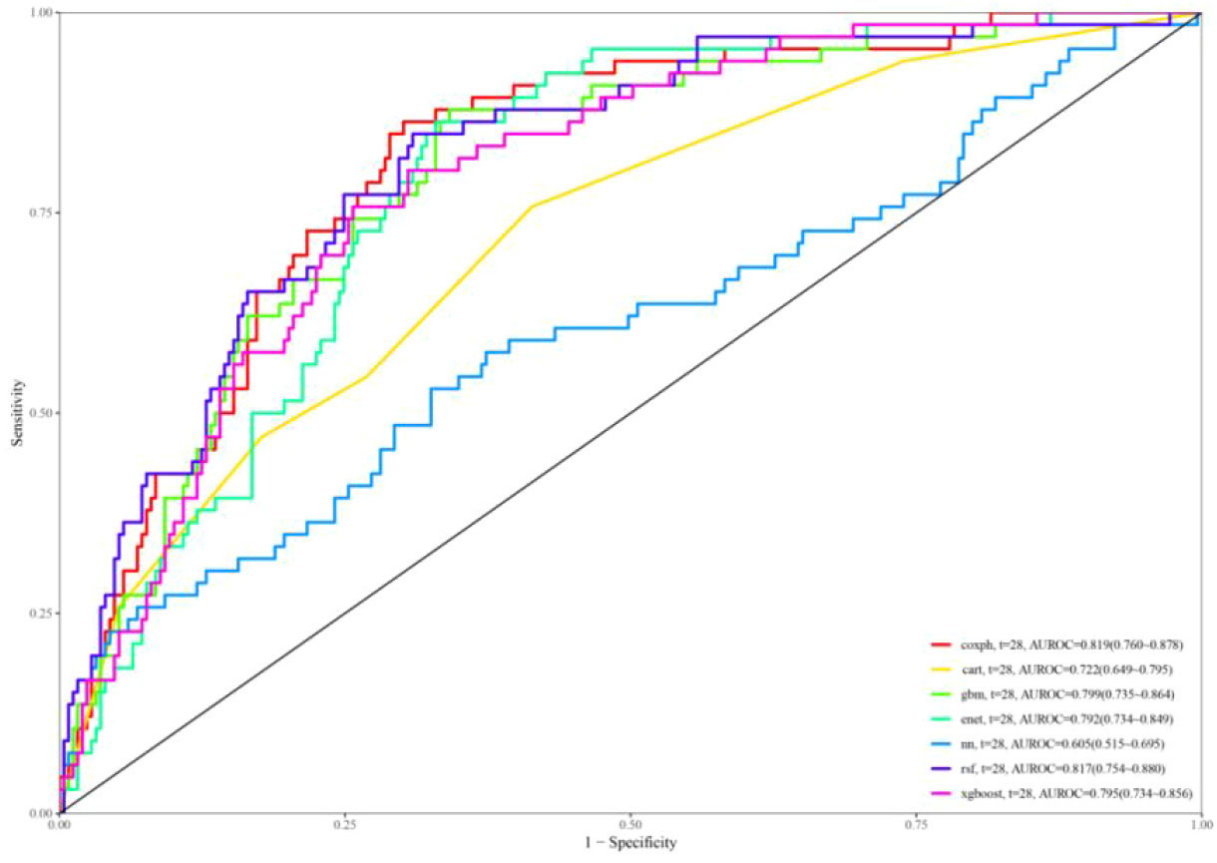
ROC curves for predicting all-cause mortality.

In **Figure 6**, the ROC curves for different models are displayed, with model performance indicated by AUC values. Coxph has an AUC of 0.819, cart has an AUC of 0.722, gbm has an AUC of 0.799, enet has an AUC of 0.792, nn has an AUC of 0.605, rsf has an AUC of 0.817, and xgboost has an AUC of 0.795. Calibration curves for each model are presented in **Supplementary Figure S1**.

The calibration curves of the coxph, dt, deepsurv, rsf, and xgboost models closely aligned with the reference line, demonstrating their strong predictive capabilities. The DCA curves (**Supplementary Figure S2**) additionally showed a significant net benefit for each model, emphasizing their robust clinical validity. Based on the decision curve and the AUC value reaching 0.817, the model established by the RSF algorithm demonstrated the best performance.

## Discussion

The simultaneous occurrence of CHF and DM poses a substantial societal health burden. Nonetheless, pinpointing straightforward and potent biomarkers to forecast these conditions is challenging. Our research included 1046 individuals with both conditions. We determined the 28-day hospital fatality rate to be 21%. The 28-day ICU fatality rate was 22%. Upon accounting for possible confounding elements, a notable positive affiliation emerged linking the TyG index and the 28-day hospital mortality (HR=1.31, 95% CI: 1.09–1.57, P=0.004) and 28-day ICU mortality (HR=1.29, 95% CI: 1.07–1.54, P=0.006). The RCS analysis indicated a linear correlation linking the TyG index and both fatality types. Consequently, TyG is a separate risk factor. It affects patients suffering from CHF and DM.

The TyG index is a blend of TG and FBG. It has become a possible predictor for metabolic disorders, atherosclerosis, and cardiovascular diseases (20–22). In hypertension patients, a higher TyG index is tied to higher fatality risk (23). A study by Yang et al. (24) bolstered the TyG index. It is a marker for unfavorable cardiovascular outcomes in chronic coronary syndrome patients. Research involving 1226 participants showed that an elevated TyG index implicated in an greater likelihood of stroke recurrence and death (25). More importantly, a study found a connection linking an elevated TyG index and worse outcomes in acute decompensated heart failure (ADHF) patients (26). Yang et al. also determined that the TyG index can predict both hospitalization and ICU mortality following a cardiac arrest (27). For those with CAD, the TyG index might assist in forecasting adverse cardiovascular events (28, 29). In critically ill patients, a one-unit rise in the TyG index implicated in a nearly 30% or greater rise in the risk of hospital mortality (30, 31). These results corroborate the material role of the TyG index in assessing patient prognosis.

An interesting finding in our study is that younger patients in the high TyG group faced a greater risk of death. This result may be explained by the presence of more severe metabolic disturbances, such as IR, in younger patients (32), which could accelerate the progression of heart failure (33). Younger patients might also have undiagnosed or poorly controlled diabetes, exacerbating heart failure (34). Moreover, lifestyle elements, like diet and exercise, might have an impact (35). These findings suggest that high TyG levels in younger patients may indicate more severe IR, which can lead to worse outcomes despite their younger age. Further studies are needed to explore these relationships in more detail.

Earlier disquisition has authenticated that the TyG index is more sensitive in detecting IR. It is also more specific than the high insulin-positive glucose clamp technique (36). IR leads to reduced insulin sensitivity, causing persistent hyperglycemia and higher glycosylation levels. This promotes collagen buildup and chronic fibrosis in heart tissue, eventually harming cardiac function (37). IR also causes chronic hyperglycemia and dyslipidemia, leading to more oxidative stress. This stress triggers inflammation, leading to foam cell formation, blood vessel damage, and accelerated smooth muscle cell proliferation (38). IR is linked to metabolic issues and inflammation, with higher levels of proinflammatory cytokines, adipokines, and catecholamines. These factors cause mild inflammation and chronic hypercatecholinemia, affecting quality of life and harming cardiac function (39). IR promotes cardiovascular disease development through mechanisms that include increasing vascular stiffness and reducing nitric oxide(NO) bioavailability (40). Furthermore, IR can trigger a harmful neurovascular cycle through humoral activation. This occurs by increasing sympathetic excitability and adrenaline release, which subsequently lead to vasoconstriction and platelet aggregation. In extreme cases, it may even result in vascular stenosis (41, 42). IR interacts with multiple pathways in CHF patients with DM, significantly increasing mortality risk.

We probed the tie connecting the TyG index and fatality in CHF and DM, developing a machine learning-based prediction model. Our findings indicated the TyG index links to CHF and DM prognosis. Higher TyG values are significantly implicated in a greater risk of death. Given the high prevalence of diabetes in CHF patients, early identification of high-risk groups is essential. As a straightforward indicator, the TyG index can effectively represent IR. It can effectively aid in evaluating patients’ long-term outcomes. Additionally, machine learning models integrating the TyG index with other clinical variables significantly enhance prognostic predictions, offering a foundation for early intervention and personalized treatment strategies. This study provides valuable support for managing CHF patients with diabetes in clinical practice and offers insights into the future use of machine learning-based prediction tools in cardiovascular medicine.

Several limitations should be conceded regarding this study. Firstly, as a single-center retrospective study, it cannot establish causality and needs further validation through a multicenter prospective study. Secondly, while medication use can influence patient outcomes, we were unable to gather comprehensive data on medication use due to database limitations. Thirdly, The study concentrated exclusively on the predictive power of the initial TyG index. However, due to the inherent constraints of the MIMIC database, discharge TyG data were not consistently available for all patients. This limitation restricted our ability to examine variations in the TyG index throughout the hospital stay and subsequent follow-up. Consequently, this omission may affect the comprehensiveness of the analysis. Fluctuations in the TyG index during hospitalization might have offered significant understanding of the evolving connection linking the TyG index and patient outcomes. Lastly, due to the inherent constraints of the MIMIC database, we could not perform a comparative analysis of the TyG index versus other indices of IR, such as HOMA-IR and QUICKI. Future studies should strive to encompass a wider array of IR indicators to offer a more thorough evaluation of their prognostic capabilities in individuals with CHF and DM.

## Conclusion

Our investigation reveals that an augmented TyG index is appreciably connected with heightened hospital and ICU fatality rates in individuals with CHF and DM. The TyG index has the capacity to predict malevolent outcomes. Importantly, the TyG index is an inexpensive, readily available marker that may improve risk stratification for patients with CHF and DM. Nonetheless, Further multicenter trials with a forward-looking design are necessary to substantiate these observations.

## Data Availability

The data that support the findings of this study are available from the MIMIC database. The researchers accessed the database using certificate number 13874481. The original contributions presented in the study are included in the article/**Supplementary Material**. Further inquiries can be directed to the corresponding author.
