# Supplementary material for: Triglyceride-glucose index and mortality in congestive heart failure with diabetes: a machine learning predictive model
Source: Front Endocrinol (Lausanne). 2025 Oct 15;16:1675152. doi: 10.3389/fendo.2025.1675152 (PMC12568326; doi:10.3389/fendo.2025.1675152)
Supplement: Supplementary file 1 [file DataSheet1.doc]

Table S1 Missing values in variables.

| Variable | Missing Value Proportion (%) |
| --- | --- |
| Weight | 16.8260038 |
| PTT | 8.3173996 |
| INR | 8.2217973 |
| PT | 8.2217973 |
| Urine output | 5.2581262 |
| Calcium | 3.8240918 |
| WBC | 1.1472275 |
| RBC | 0.9560229 |
| Platelet | 0.7648184 |
| Sodium | 0.5736138 |
| Potassium | 0.4780115 |
| BUN | 0.4780115 |
| Temperature | 0.3824092 |
| GCS | 0.3824092 |
| SOFA | 0.2868069 |
| Creatinine | 0.2868069 |


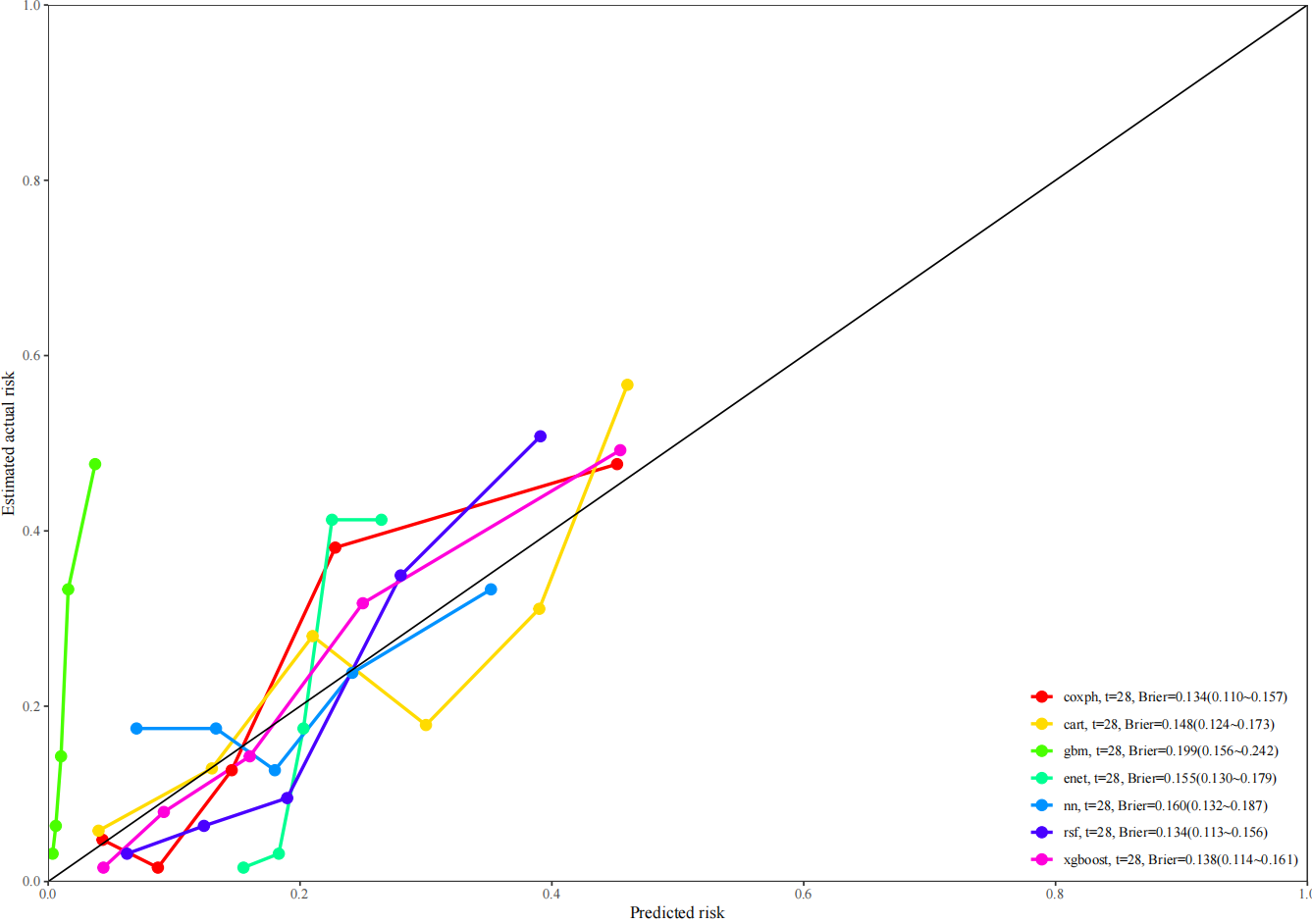


Figure S1 Calibration Curve.


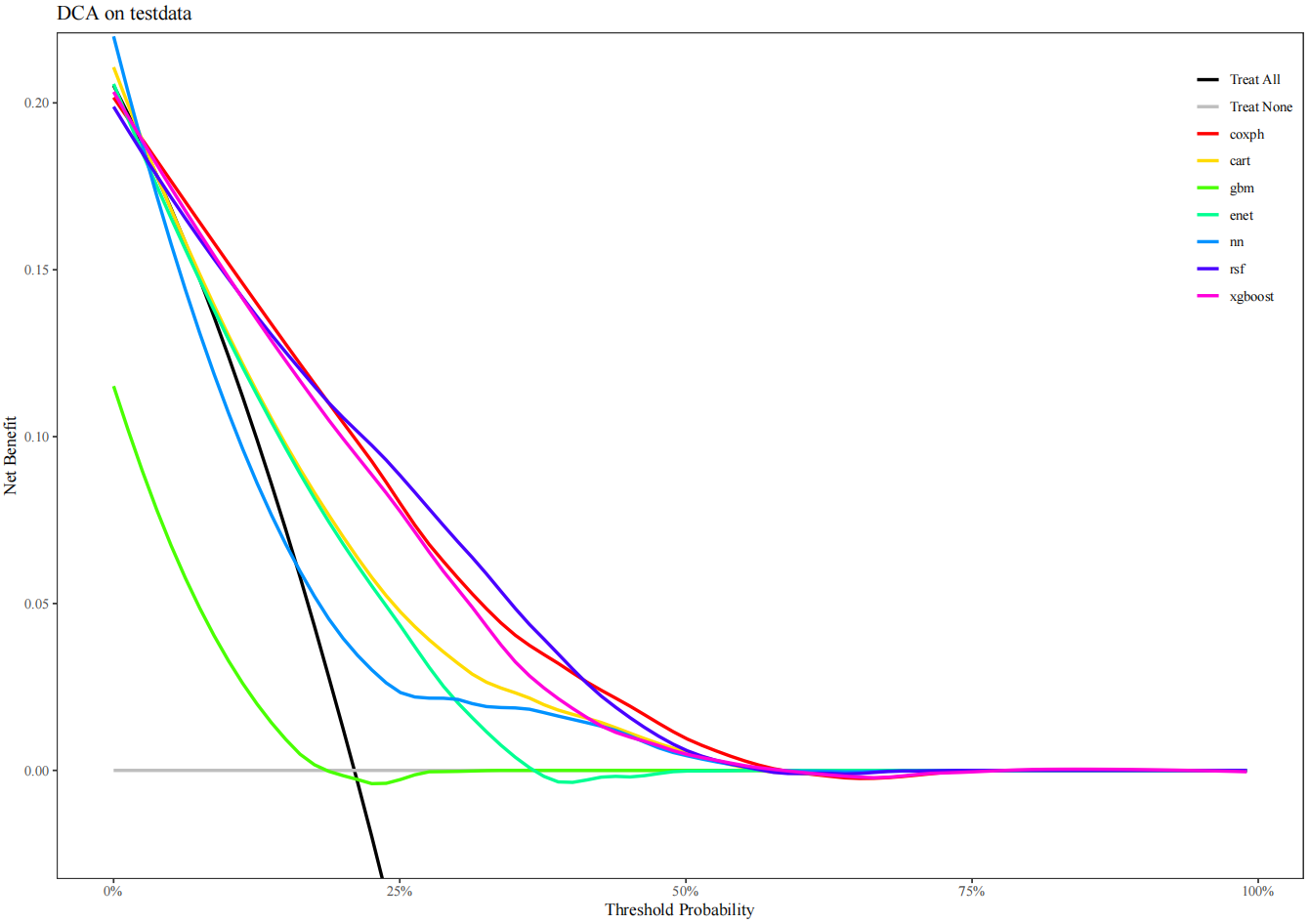


Figure S2 DCA curve.
